# Mesoporous silica nanoparticles loaded Au nanodots: a self-amplifying immunotherapeutic depot for photothermal immunotherapy

**DOI:** 10.3389/fimmu.2025.1616539

**Published:** 2025-06-18

**Authors:** Chuan Wang, Dongmei Wang, Liang Huang, Qi An

**Affiliations:** Scientific Reasearch and Teaching Department, Public Health Clinical Center of Chengdu & Public Health Clinical Center of Chengdu University of Traditional Chinese Medicine, Chengdu, Sichuan, China

**Keywords:** Au nanodots, mesoporous silica nanoparticles, photothermal immunotherapy, immunogenic cell death, T-cell infiltration

## Abstract

**Introduction:**

Photothermal therapy (PTT) has emerged as a highly promising approach for cancer treatment due to its advantages of localized treatment, controllable irradiation, and non-invasive nature. This study presents a multifunctional platform for tumor PTT based on Au nanoparticles-decorated mesoporous silica nanoparticles (MSN@Au), aiming to synergize photothermal ablation with immune modulation.

**Methods:**

MSN@Au nanoparticles were engineered as the PTT agent. Photothermal efficiency was evaluated under 808 nm near-infrared (NIR) laser irradiation. Anti-tumor efficacy was systematically assessed both in vitro (tumor cell cultures) and in vivo (tumor-bearing animal models). Immune responses were analyzed by examining immunogenic cell death (ICD) induction, dendritic cell maturation, and cytotoxic T cell activation/infiltration within the tumor microenvironment (TME).

**Results:**

MSN@Au demonstrated exceptional photothermal conversion efficiency under NIR irradiation, leading to significant tumor cell inhibition in both *in vitro* and *in vivo*. Mild PTT mediated by MSN@Au not only caused direct tumor cell damage but also triggered ICD. This promoted dendritic cell maturation and enhanced activation/infiltration of cytotoxic T cells within the TME, thereby amplifying anti-tumor immunity.

**Discussion:**

This study underscores that the strategic design of MSN@Au as a PTT agent successfully induces ICD while modulating the immunosuppressive TME, significantly amplifying therapeutic efficacy. The integration of efficient photothermal ablation with immune activation opens new avenues for developing next-generation nanoplatforms that synergize PTT with immune modulation, offering a promising strategy for treating solid tumors.

## Introduction

Cancer remains a significant global health challenge, with high mortality rates and complex treatment hurdles. Traditional therapies, such as chemotherapy, often fail to effectively eradicate tumors due to their inherent complexity, heterogeneity, and the development of drug resistance. In response to these limitations, numerous innovative therapeutic strategies have been explored to enhance treatment efficacy. Among these, photothermal therapy (PTT) has gained significant attention due to its precision, targeted approach, controlled energy delivery, and minimally invasive nature, positioning it as a promising alternative in cancer treatment (1–3).

Mild PTT, typically conducted at a moderate temperature of approximately 42-43°C, facilitates the enhanced accumulation and deep tissue penetration of photothermal nanoagents (4). Beyond its direct thermal effects, mild PTT is increasingly recognized as a critical modality for inducing ICD, a form of cell death that activates specific immune responses against tumor cells (5, 6). ICD plays a pivotal role in reshaping the tumor microenvironment (TME), converting it from a “cold” to a “hot” tumor, thus enhancing anti-tumor immunity. During ICD, tumor cells release or express various damage-associated molecular patterns (DAMPs), such as adenosine triphosphate (ATP), high mobility group box 1 protein (HMGB1), and calreticulin (CRT), which are essential for dendritic cell (DC) maturation (7). These DAMPs subsequently promote the infiltration of cytotoxic T cells into the tumor, amplifying the immune response.

Metal nanoparticles (NPs), particularly those exhibiting localized surface plasmon resonance (LSPR), are emerging as highly promising candidates for PTT due to their unique optical and thermal properties (8, 9). Among these, Au NPs have garnered considerable attention for their exceptional photothermal performance, biocompatibility, and low toxicity (10–13). Au-based nanostructures, including nanodots, nanoraspberries, nanorods, and nanocrystals, have been successfully synthesized (14–17). Au nanodots, in particular, stand out due to their size-dependent properties, high surface area, stability, and tunable characteristics, which enhance their biological interactions and photothermal conversion efficiency (14, 15, 17, 18). Small Au nanodots also exhibit superior biocompatibility and reduced long-term toxicity compared to larger counterparts, as they are more efficiently cleared via renal pathways post-treatment. While larger nanostructures may offer stronger absorption, their prolonged retention in reticuloendothelial organs (e.g., liver, spleen) raises safety concerns in clinical translation (19). These features make them well-suited for advanced biomedical applications, including diagnostics, imaging, and targeted therapy (20, 21). However, one major limitation of Au nanodots is their rapid renal excretion, which can reduce their efficacy and limit their medical applicability (10). Therefore, developing an efficient drug delivery system for Au nanodots is essential to overcoming this challenge.

Mesoporous silica nanoparticles (MSNs) provide an excellent platform for drug delivery due to their high drug loading capacity, tunable properties, controlled release capabilities, biocompatibility, and enhanced cellular uptake (22, 23). MSNs also offer remarkable stability and versatility in a wide range of biomedical applications, making them an ideal choice for integrating with Au nanodots to improve their therapeutic efficacy (24).

**Scheme 1:**
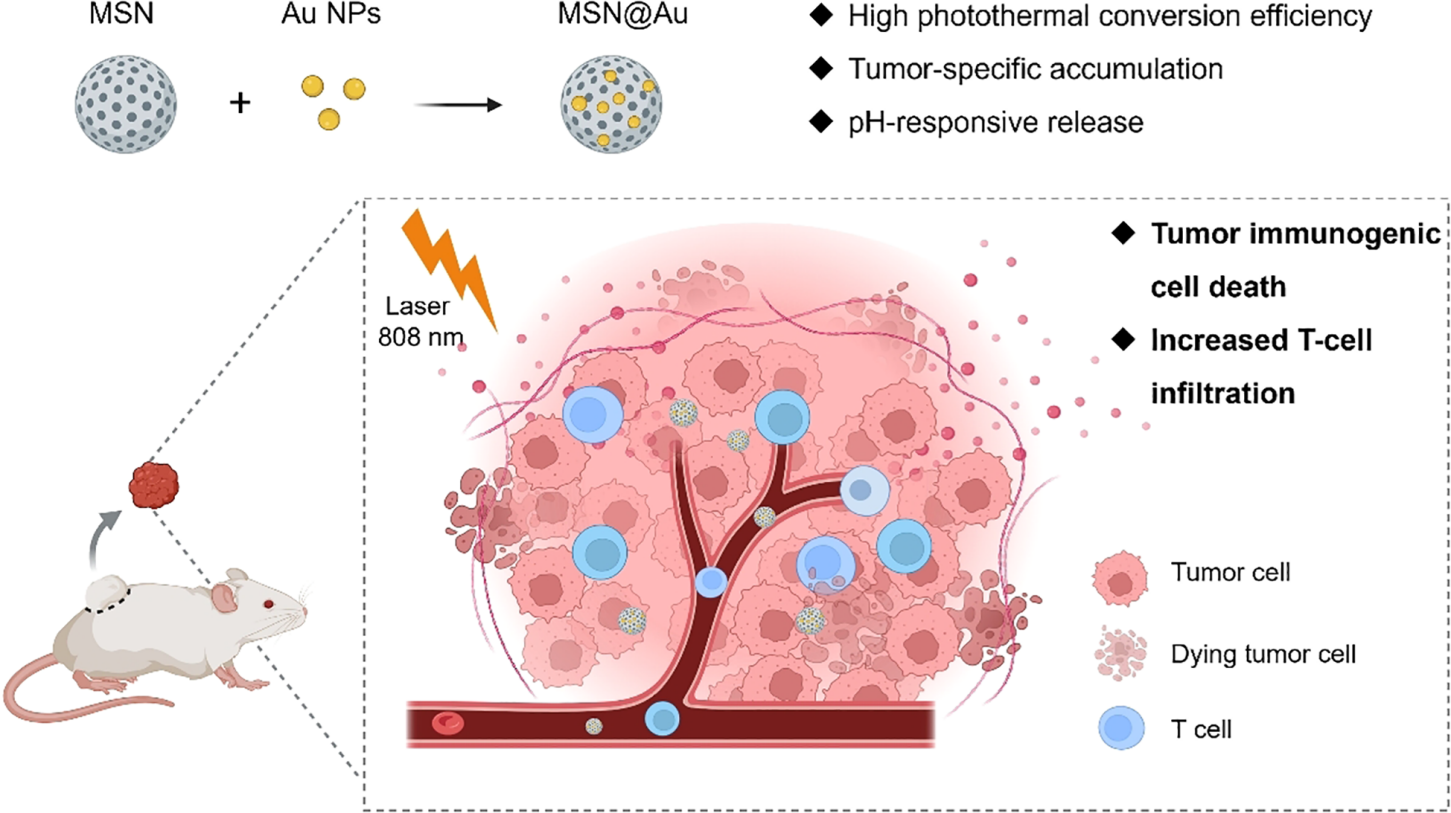
Schematic diagram of the photothermal immunotherapy with MSN@Au.

In this study, we propose a novel approach to enhance PTT efficacy by decorating Au nanodots onto MSNs, forming a composite system designated as MSN@Au (**Scheme 1**). This hybrid structure not only preserves the high photothermal conversion efficiency of Au but also significantly enhances the therapeutic efficacy under near-infrared (NIR) irradiation, effectively killing tumor cells *in vitro* and *in vivo*. Additionally, MSN@Au demonstrates remarkable capabilities in promoting ICD, facilitating DC maturation, and enriching cytotoxic T cells. These immune responses are pivotal for overcoming the immunosuppressive TME, enabling enhanced anti-tumor immunity. Importantly, a single dose of MSN@Au results in excellent anti-tumor efficacy with minimal systemic toxicity, highlighting its potential as an effective and safe therapeutic platform. The multifunctional properties of MSN@Au present a compelling paradigm for the design and synthesis of next-generation photothermal immunotherapeutic agents. By combining the benefits of PTT and immune modulation, this platform offers a promising strategy for advancing cancer treatment and furthering the development of nanomedicine-based therapies.

## Materials and methods

### Experimental reagents

Tetraethyl orthosilicate (TEOS; >99%, GC), brominated hexadecanediol (99%), urea (AR, 99%), cyclohexane (AR, 99%), n-butanol (99%) were purchased from Shanghai Macklin Biochemical Co. Ltd (China). All the reagents were of analytical grade. Fetal bovine serum (FBS) was obtained from BI (Biological Industries, Israel). APC-Cyanine7 Anti-Mouse CD11c (N418), PE Anti-Mouse CD80 (16-10A1), APC Anti-Mouse CD86 (GL-1), FITC Anti-Mouse MHC Class I (M5/114.15.2) were obtained from Tonbo Biosciences (USA).

### Preparation of MSN@Au

Preparation of MSNs: 2 g of brominated hexadecanediol (CPB), 1.2 g of urea, and 30 mL of ultrapure water are first combined; then, 30 mL of cyclohexane and 1.1 mL of n-butanol are added. The mixture is continuously stirred at 200 rpm while 5 mL of tetraethyl orthosilicate (TEOS) is added dropwise, followed by stirring at room temperature for 30 minutes. Next, the reaction mixture is transferred to an oil bath at 70°C for 1 hour, then to an ice water bath at 10°C for 2 hours, and finally back to the oil bath at 70°C for 12 hours. After the reaction is complete, the product is centrifuged (15,000 g for 20 minutes) and washed three times with ethanol, and the final MSNs were dispersed in ethanol for long-term storage.

Synthesis of Au NPs: AuNPs were synthesized according to the procedures described in our previous work (25). Fix a three-neck round-bottom flask on a heating mantle. Add 99 mL of ultrapure water into the flask under magnetic stirring (500 rpm). Attach a reflux condenser to one neck to prevent solvent evaporation. Slowly add 1 mL of 1% (w/v) chloroauric acid (HAuCl_4_) solution through a second neck while stirring. Heat the mixture to boiling (100°C) and maintain gentle boiling for 5 minutes. Rapidly inject 10 mL of 1% (w/v) sodium citrate solution into the boiling mixture via a syringe. Observe an immediate color transition from pale yellow to wine red, indicating the formation of colloidal Au NPs. Continue refluxing the solution for 20 minutes to ensure complete reduction and particle stabilization. Turn off the heating mantle and allow the solution to cool naturally to room temperature. Store the Au nanoparticle suspension at 4°C for further characterization.

Preparation of Au-PEI: 0.4 g of polyethyleneimine (PEI) was rapidly added to a 50 mL aqueous suspension of Au nanoparticle. The mixture was vigorously stirred at room temperature for 30 minutes to obtain a homogeneous aqueous solution. Subsequently, the resulting mixture was then dialyzed (MWCO = 100,000 Da) against water for 24 hours to remove unreacted precursors and impurities, yielding the final Au-PEI product. The purified Au-PEI was centrifuged at 16,000 g for 15 minutes and stored at 4°C for subsequent experiments.

Preparation of MSN-Au: A mixture of Au-PEI (1 mg) and MSN (10 mg in water) was stirred overnight at room temperature. MSH-Au was obtained after centrifugation at 15,000 g for 20 min. Supernatants were removed, MSH-Au was washed twice with water. For long-term storage, MSH-Au was dispersed in ethanol.

### Characterization of MSN@Au

The morphology and size of Au and MSN@Au were detected by transmission electron microscopy (JEOL JEM-F200 field emission TEM). Zeta potential and DLS measurements were performed with a Malvern Zetasizer Nano ZS90 (Malvern, UK). The changes in the solution temperature were measured with an OM-CPQuadTemp2000 temperature recorder. Thermal images were achieved with an infrared thermal imaging system.

### *In vitro* photothermal properties of MSN@Au

The photothermal performance of MSN@Au at different concentrations and under varying power densities was evaluated. Suspensions of MSN@Au (32, 128, 512 μg/mL in deionized water, prepared via 5-minute ultrasonication to ensure homogeneity) were loaded into 1.5 mL Eppendorf tubes with sample volume of 1.0 mL per tube. These samples were subjected to near-infrared (NIR) laser irradiation using a VA-I-DC-808 laser system (Beijing Viasho Technology Co., Ltd., China) operating at a wavelength of 808 nm with a calibrated power density of 1 W/cm². Temperature monitoring was conducted in real time using an AI-518P high-precision temperature controller (YUDIAN Automation Technology Co., Ltd., China). While maintaining the concentration of MSN@Au at 32 μg/mL, we irradiated the samples with laser power densities of 1.0, 1.5, and 2.0 W/cm², respectively, and recorded the temperature elevation profiles.

### *In vitro* cytotoxicity

The human breast carcinoma cell line MDAMB-231 and 4T1 murine breast cell line was purchased from the Cell Bank of the Chinese Academy of Science (Shanghai, China). The MDAMB-231 cells were cultured in Dulbecco’s modified Eagle’s medium (DMEM) (Wisent Inc.) and the 4T1 cells were maintained in RPMI 1640 medium (Wisent Inc.). The cell culture media were supplemented with 10% fetal bovine serum (FBS) (Gibco) and 100 U/mL penicillin/streptomycin (Invitrogen). The cells were incubated at 37°C in a humidified environment with 5% CO_2_.

Cell Counting Kit-8 (CCK-8) (Dojindo, Japan) was applied to measure the cell viability after different treatments. Briefly, 5000 4T1 cells per well were seeded in 96-well plates incubated overnight. Then the cells were treated with Au NPs, MSN, MSN@Au (20 μg/mL Au). After 6 h incubation, the cells were exposed to PTT (808 nm laser with 0.5 W cm ^−2^ for 5 min) treatment. With another 24 h incubation, the medium was removed and 100 μL of media containing CCK-8 (10%) was added and incubated at 37°C for 2 h, the absorbance at 450 nm was measured using a multifunctional microplate reader (Infinite 200, TECAN). Cell viability of untreated 4T1 cells was considered as 100%. Each sample was triplicated, and data represented the mean value of all measurements. In addition, cells incubated with the same concentration of these nanomaterials without NIR light irradiation were also used as comparison. Besides CCK-8 assay, we used calceinAM and PI to stain the cells after different treatments. The green and red fluorescence was observed by an inverted fluorescence microscope (Leica).

A reactive oxygen species (ROS)-sensitive probe, sensitive probe, 2’,7’-dichlorodihydrofluorescein diacetate (DCFH-DA), which could be oxidized to the highly fluorescent DCF by ROS, was chosen to detect the intracellular generation of ROS. After a 6 h incubation with MSN@Au (20 μg/mL Au), 4T1 cells were washed with PBS to remove uninternalized nanoparticles. Fresh culture media containing DCFH-DA (20 µM) was then added, followed by a 20 min incubation at 37°C in the dark. DCFH-DA was prepared by dissolving in anhydrous DMSO (1 mM stock) and diluting in serum-free medium. During this period, intracellular esterases hydrolyzed DCFH-DA to non-fluorescent DCFH, which is oxidized to fluorescent dichlorofluorescein (DCF) in the presence of reactive oxygen species (ROS). Post-incubation, cells were washed again with PBS to remove residual probe and resuspended in phenol red-free medium to minimize background interference. Laser scanning confocal microscopy (Zeiss LSM 880, excitation/emission: 488 nm/525 nm) was employed to capture images of DCF fluorescence (green). For quantitative analysis, cells were trypsinized, centrifuged at 300 g for 5 min, and resuspended in PBS. Fluorescence intensity was measured using a BD Accuri C6 flow cytometer (excitation: 488 nm; emission filter: 530/30 nm), with a minimum of 10,000 events recorded per sample.

### *In vivo* blood half-life and biodistribution measurement

Au NPs and MSN@Au NPs (5 mg/kg, Au) were intravenously injected in BALB/c mice (4–6 weeks, 20 g of body weight). Blood samples were collected at 2 min, 5 min, 0.5 h, 3 h, 6 h, and 24 h post injection, respectively, and stored at −20°C before ICP-MS analysis. Each group has four mice as replicates. After the quantitative measurements by ICP-MS, the data were analyzed and evaluated. The pharmacokinetic data of the AuNPs and MSN@Au NPs were fitted into the classical two-compartment pharmacokinetic model. The absorption half-life (t1/2a) and elimination half-life (t1/2b) were calculated. At 24 h, tumors and major organs (heart, liver, spleen, lung, kidney) were collected and determined by ICP-MS. The Au content was expressed as % ID/g.

### *In vivo* anticancer efficacy evaluation

The animal study was approved by The Animal Ethics Committee of Chengdu University of Traditional Chinese Medicine. The study was conducted in accordance with the local legislation and institutional requirements. Six-week-old female BALB/c mice were purchased from the Beijing Vital River Company (Beijing, China). The 4T1 tumor models were successfully established by subcutaneous injection of 1×10^5^ cells suspended in 100 µL PBS into the right back of each mouse. The mice were treated when the tumor volumes approached 100 mm^3^. The mice were randomly distributed into four groups (n = 5, each group), and 100 µL of the different NPs was intravenously injected into the tumor-bearing mice: 1) control group (only received PBS); 2) MSN@Au administrated only; 3) Au administrated + NIR irradiated with laser 808 nm; 4) MSN@Au administrated + NIR irradiated with laser 808 nm. All the groups received only one injection. The concentration of Au = 5 mg/kg. For NIR irradiated groups, the tumors were exposed to 808 nm laser at 1 W cm^−2^ for 5 min at 6 h post-injection, respectively. The therapeutic results of each group were evaluated by measuring the tumor volumes the other day. Tumor volume = length × width 2/2. The temperature changes of the tumors after injection at different time was recorded by an IR thermal imaging system when the tumors were exposed to 808 nm laser light.

On day 7, lymph nodes adjacent to the tumors were excised. These excised lymph nodes were subsequently subjected to enzymatic digestion using an RPMI 1640 medium supplemented with 0.5 mg/mL of collagenase IV and 0.1 mg/mL of DNase I, incubated at 37°C for a duration of 30 minutes. Following digestion, the released cells were passed through a 40 μm cell strainer to obtain a single-cell suspension. The isolated lymph node cells were then washed in RPMI 1640 medium before being stained with specific antibodies: anti-CD11c (at a concentration of 0.25 µg per 100 µL), anti-CD80 (1.0 µg per 100 µL), and anti-CD86 (0.25 µg per 100 µL) for one hour under ice-cold conditions. Finally, flow cytometric analysis was performed on the stained cellular samples.

After 28 d from the therapy, the major organs (liver, lung, kidney, heart, and spleen) from control and treated mice were excised, fixed in 4% paraformaldehyde (PFA) solution and then processed routinely into paraffin. The sliced major organs were stained with H&E and examined by an inverted florescence microscope system (Nikon-Ti-S). Tumors were collected and weighed. The tumors from different groups were collected and fixed in 4% PFA for immunofluorescence staining.

## Results and discussion

**Figure 1:**
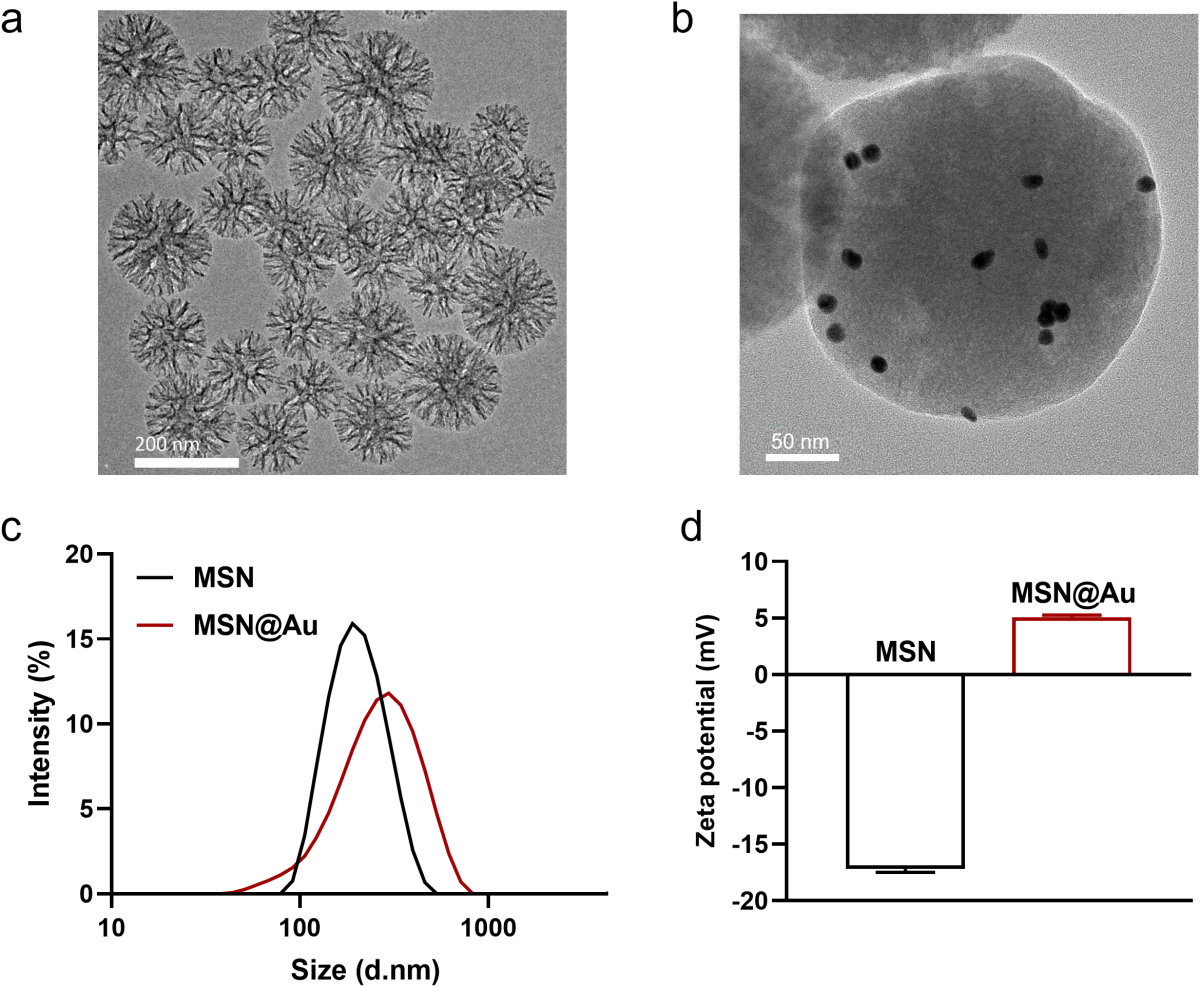
Characterization of MSN@Au. **(a, b)** TEM images of MSN and MSN@Au. **(c)** Hydrodynamic diameter of MSN and MSN@Au. **(d)** Zeta potential of MSN and MSN@Au.

Firstly, MSNs were synthesized, followed by immobilization of polyethyleneimine (PEI, MW = 2.5 k)-functionalized Au NPs onto the MSN surfaces via electrostatic interactions. Au NPs exhibited an average particle size of approximately 13 nm, with surface charge reversal observed following PEI functionalization (**Supplementary Figure 1**). As revealed by transmission electron microscopy (TEM) results, the synthesized MSNs exhibited an ordered mesoporous structure (**Figure 1a**). After loading, Au NP were sparsely distributed on the MSN surfaces (**Figure 1b**). Dynamic Light Scattering (DLS) measurements demonstrated excellent monodispersity of the NPs both before and after Au NPs deposition (**Figure 1c**). Following Au NPs functionalization, the hydrodynamic diameter of the particles increased from 188.9 nm to 249.6 nm, while the surface zeta potential shifted from -16.7 mV to + 4.8 mV (**Figure 1d**). Element mapping also confirmed the successful incorporation of Au NPs through clear colocalization of Au and Si signals (**Supplementary Figure 2**). These changes in particle size and surface charge are critical for subsequent biomedical applications, as they may influence nanoparticle stability, cellular uptake efficiency, and biodistribution within biological systems (26–28). In addition, MSN@Au exhibited high stability in PBS solutions, as demonstrated that the hydrodynamic diameters of MSN@Au on day 0 and day 7 were 249.6 and 258.0 nm, respectively (**Supplementary Figure 3**).

**Figure 2:**
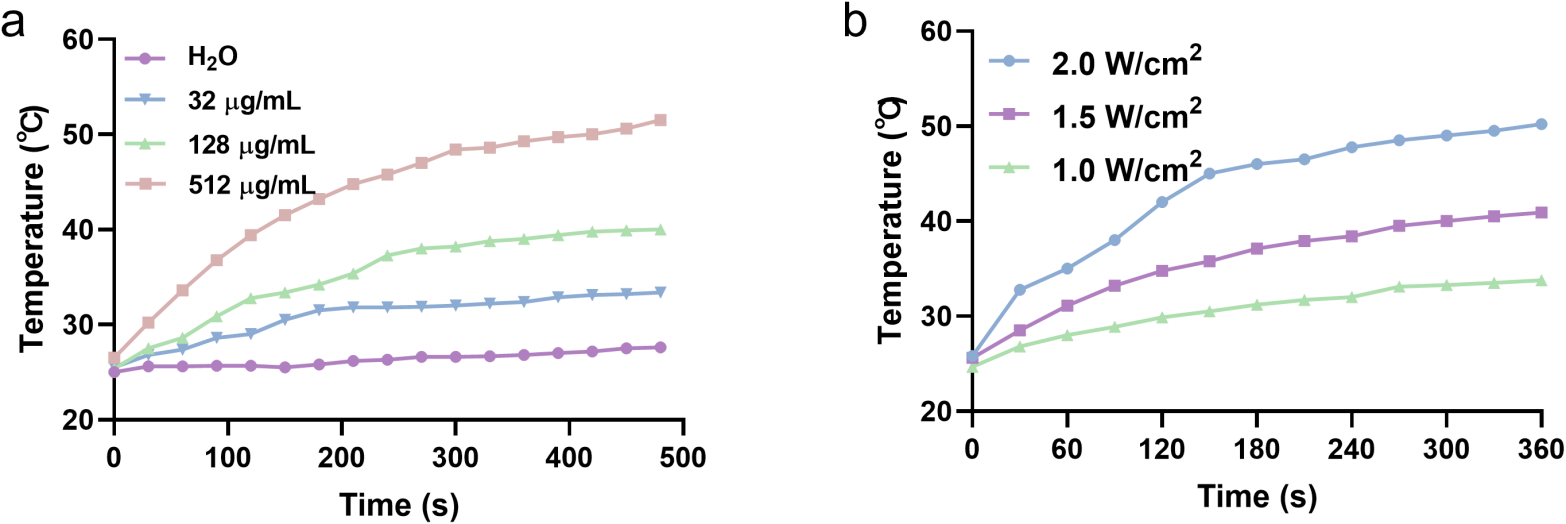
Photothermal property of MSN@Au. **(a)** Concentration-dependent temperature changes of MSN@Au during 808nm laser irradiation (1W/cm^2^). **(b)** The photothermal heating curves of MSN@Au at different power densities with 808 nm laser irradiation. The concentration of MSN@Au is 32 μg/ml.

Subsequently, the absorption profiles of Au NPs and MSN-Au were measured. There was a small change in the optical properties of AuNP after loading on mesoporous silica, where a slight shift in absorbance maxima (λspr) was observed (**Supplementary Figure 4**). This enhanced NIR absorption compared to bare AuNRs attributed to silica loading-induced plasmonic coupling. The photothermal conversion capabilities of various MSN@Au nanocomposites were systematically investigated under near-infrared (NIR) laser irradiation. As illustrated in **Figure 2**, all MSN@Au solutions demonstrated significant temperature elevation upon NIR exposure, with the temperature profiles exhibiting distinct temporal progression. Notably, the photothermal response of MSN@Au manifested dual dependency characteristics: a pronounced concentration-dependent relationship (**Figure 2a**) and a marked laser power-dependent correlation (**Figure 2b**). These findings collectively validate that the photothermal performance of MSN@Au can be precisely regulated by both nanoparticle dosage and irradiation parameters, offering a tunable platform for optimizing therapeutic outcomes in site-specific antitumor applications. The dual dependency not only enhances the adaptability of MSN@Au in complex physiological environments but also minimizes off-target thermal damage through spatiotemporal control. Furthermore, quantitative analysis revealed that MSN@Au demonstrated a higher photothermal conversion efficiency (η) value (35.6% ± 1.8%) than Au NPs (31.3% ± 2.1%) (**Supplementary Figure 5**). The observed superiority of MSN-Au can be attributed to two synergistic mechanisms. First, confining Au NPs within the mesoporous channels of MSNs promotes localized plasmonic coupling between adjacent Au NPs, as previous theoretical and experimental studies have established a positive correlation between the surface electric field intensity of metallic nanoparticles and their plasmonic extinction capabilities (29–32). This coupling induces a red-shifted and intensified absorption peak in the NIR region, facilitates efficient photon absorption and subsequent thermal energy conversion, thereby contributing to its superior photothermal performance. Second, the MSN framework physically isolates Au NPs, preventing their laser-induced aggregation—a common issue with free Au NPs in solution. Aggregation typically broadens plasmonic absorption peaks and reduces photothermal stability. Therefore, the combinatorial effects of enhanced light absorption and stabilized plasmonic units collectively contribute to the PTT performance of MSN@Au.

**Figure 3:**
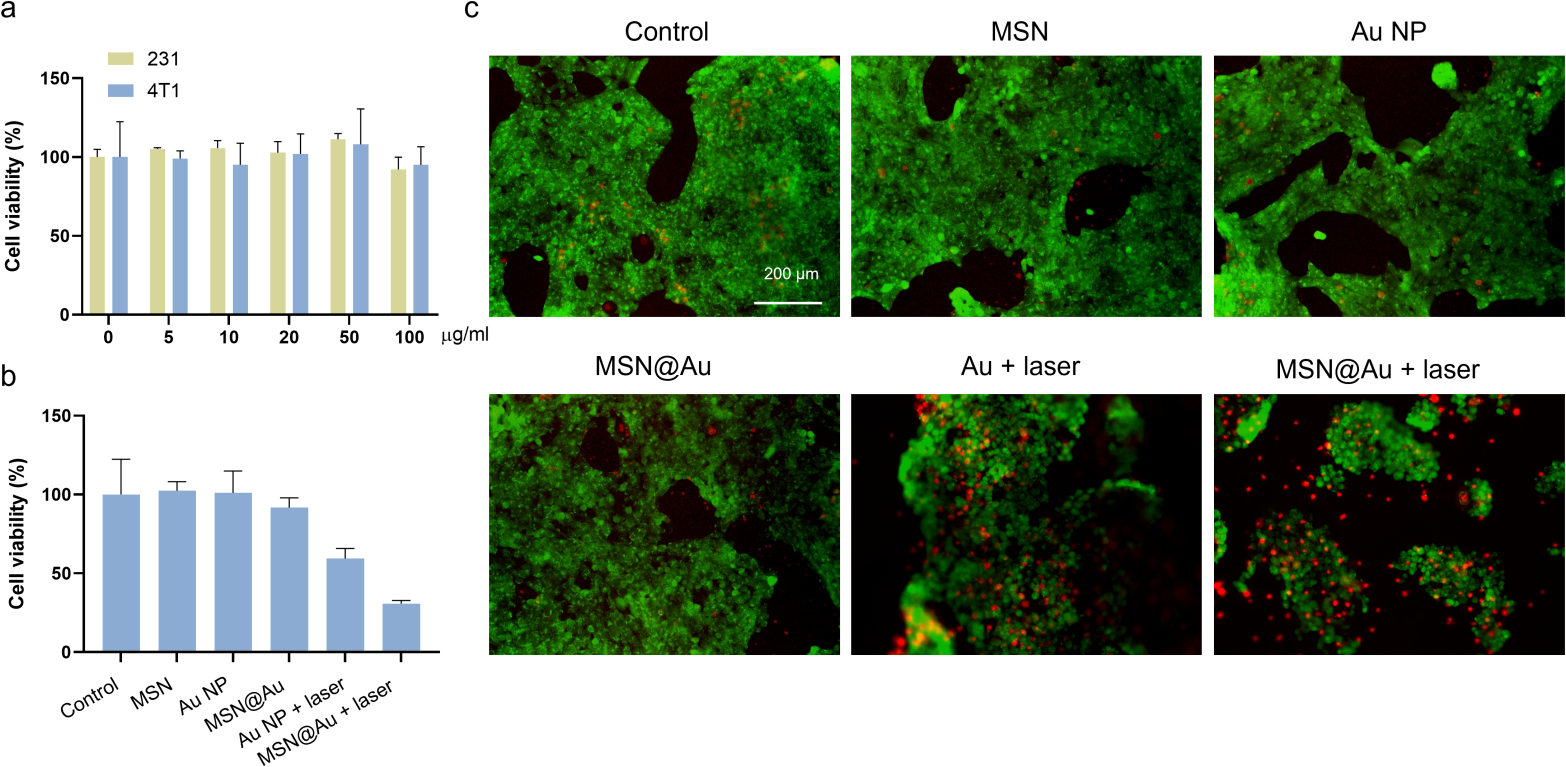
*In vitro* totoxicity. **(a)** Cytotoxicity data of MDA-MB-231and 4T1 cells treated with different concentrations of MSN@Au. **(b)** Cytotoxicity data of 4T1 cells after different treatments. **(c)** Fluorescence images of 4T1 cells after incubation with MSN, Au and MSN@Au in different irradiating conditions. The cells were stained with calcein-AM and PI. Scale bars, 200 µm. The mean ± s.d. from three independent replicates is shown. ***P < 0.001, **P < 0.01, calculated by two-way ANOVA.

To evaluate the *in vitro* cytotoxicity of Au and MSN@Au in cancer cells, a standard CCK-8 assay was performed to assess the relative cell viability under different irradiation conditions. Even at elevated concentrations of up to 100 µg/mL, MSN@Au exhibited no significant cytotoxicity against both MDA-MB-231 and 4T1 breast cancer cell lines (**Figure 3a**). As shown in **Figure 3b**, laser treatment (808 nm at 0.5 W/cm² for 5 min) led to a decrease in cell viability in both Au and MSN@Au groups; however, no notable reduction in cell viability was observed in the corresponding dark control groups. A significant reduction in cell viability (less than 50%) was observed in cells treated with the combination of MSN@Au and laser irradiation, indicating the effective PTT exerted by MSN@Au on tumor cells. To further validate these findings, we employed a live/dead staining assay using calcein-AM and propidium iodide (PI). Calcein-AM is hydrolyzed by endogenous esterases to generate green fluorescence, which indicates live cells, while PI stains dead cells with red fluorescence. As illustrated in **Figure 3c**, in the absence of laser irradiation, nearly all cells exhibited green fluorescence, suggesting that MSN, Au, or MSN@Au alone did not induce cell death. In contrast, when MSN@Au-exposed cells were irradiated with the 808 nm laser, most cells displayed red fluorescence, confirming that the photothermal effect efficiently induced cell death.

**Figure 4:**
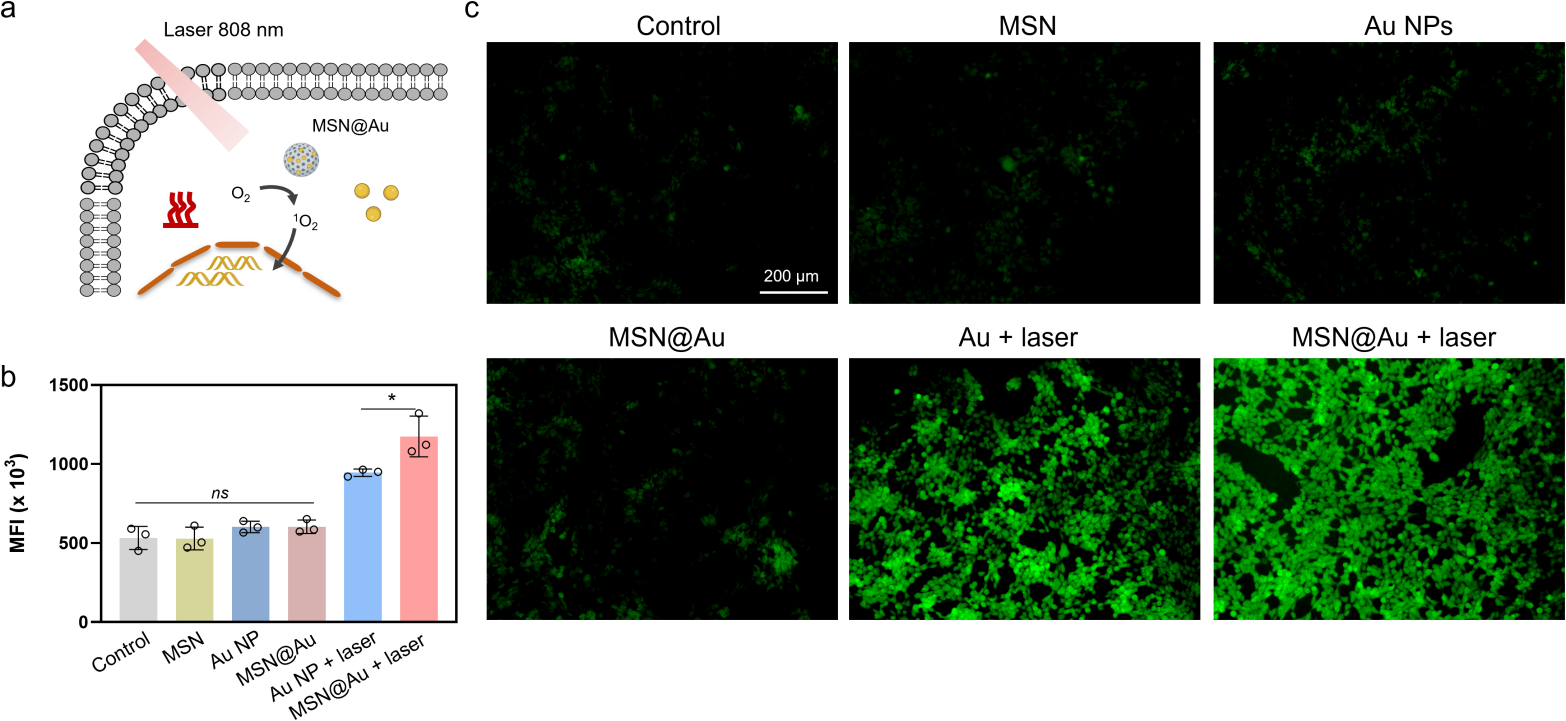
The intrinsic photodynamic performance of MSN@Au. **(a)** Schematic representation of ROS generation under light exposure. **(b, c)** The mean fluorescence intensity and fluorescence images of 4T1 cells stained with DCFH-DA. The mean ± s.d. from three independent replicates is shown. ***P < 0.001, *P < 0.05, ns represents no significant difference, calculated by two-way ANOVA.

Leveraging the intrinsic photodynamic properties of Au NPs, we systematically evaluated the light-triggered intracellular photodynamic activity across treatment groups using DCFH-DA as a ROS probe (**Figure 4a**). This fluorogenic compound generates green fluorescence proportional to intracellular ROS levels. Control groups treated with MSNs, Au NPs, or MSN@Au alone exhibited negligible ROS elevation under dark conditions. Strikingly, upon 808 nm laser irradiation (0.5 W/cm^2^, 5 min), both Au NPs and MSN@Au triggered substantial ROS generation, as evidenced by dramatically intensified DCFH fluorescence signals (**Figures 4b, c**). This synergistic enhancement highlights the optimized photodynamic efficacy of the MSN@Au hybrid system while maintaining excellent cellular biocompatibility, as demonstrated by baseline ROS levels in non-irradiated conditions. Moreover, GSH overexpression in tumor tissue adapts for high levels of oxidative stress, consuming ROS, which is considered as a major obstacle for ROS-mediated tumor therapy (33). The intracellular GSH concentration decreased more with MSN@Au + laser treatment than Au NPs (**Supplementary Figure 6**), indicating effective GSH depletion.

Pharmacokinetic profiling and biodistribution analysis serve as critical prerequisites for the targeted applications of nanomedicines (34, 35). To ensure therapeutic efficacy, it is important to prolong the blood half-life of AuNPs while enhancing their tumor-targeting specificity (17, 36). Thus, Au NPs and MSN-Au NPs (5 mg/kg, Au) were intravenously injected in BALB/c mice. Blood samples were collected at different time and measured by ICP-MS. The pharmacokinetic data of the AuNPs and MSN-Au NPs were fitted into the classical two-compartment pharmacokinetic model and the absorption half-life (t1/2α) and elimination half-life (t1/2β) were calculated. Au NPs exhibited a short α-phase half-life (6.2 min), indicating rapid clearance from the bloodstream into tissues or organs. MSN@Au showed a 3-fold longer α-phase (19.5 min), suggesting enhanced stability in circulation due to the mesoporous silica coating (**Supplementary Figure 7**). This delays uptake by the reticuloendothelial system (RES) or nonspecific tissue distribution. Furthermore, MSN@Au’s β-phase half-life (6.42 h) is 3 times longer than that of Au NPs (2.12 h), reflecting significantly prolonged systemic retention. This is attributed to reduced renal clearance and RES recognition, a common advantage of silica-coated nanostructures (37). Additionally, preferential accumulation on tumorous region, and subsequent biodistribution of MSN@Au was investigated. MSN-Au was primarily cleared through the liver and kidney, and the intratumoral Au concentration reached 7.12% ID/g when examined 24 h postinjection (**Supplementary Figure 8**).

**Figure 5:**
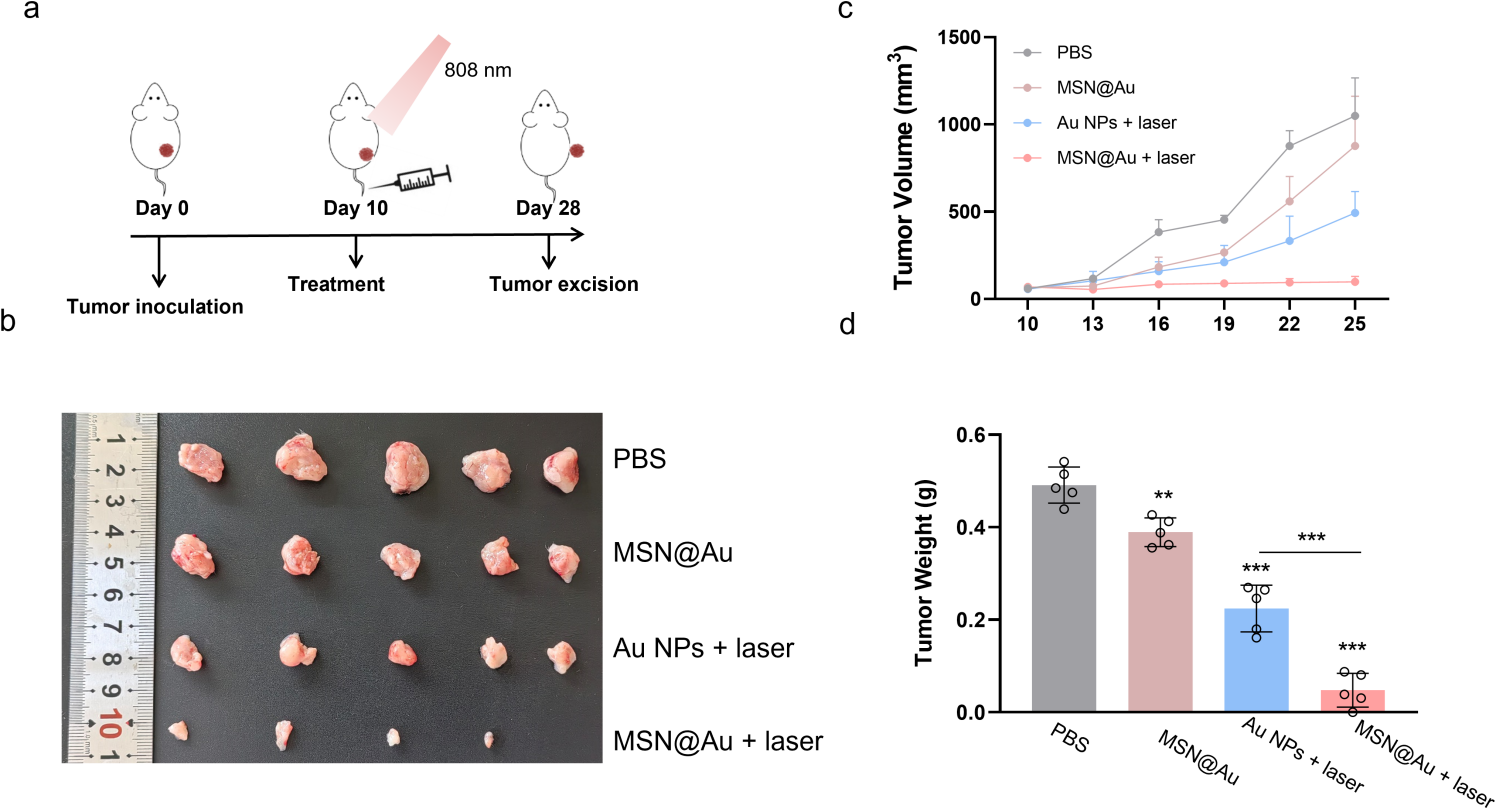
*In vivo* therapeutic efficacy of MSN@Au-PTT combined therapy. **(a)** Treatment timeline for *in vivo* anti-tumor therapy. 10^5^ 4T1 tumor cells were subcutaneously injected into BALB/c mice. When the tumor volume reached 100 mm^3^, the mice were randomly distributed into four groups (n = 5), and mice received one injection of Au (0.1 mg/kg) or MSN@Au (10 mg/kg), respectively. For NIR irradiated groups, the tumors were exposed to 808 nm laser at 1W cm^−2^ for 5 min at 6 h post-injection. **(b)** Photographs of distant tumors of different treatment groups. **(c)** Growth curves of tumors. **(d)** Weights of tumors on day 28. ***P < 0.001, **P < 0.01, calculated by one-way ANOVA.

The therapeutic efficacy of MSN@Au was evaluated in a 4T1 tumor-bearing mice following the experimental timeline shown in **Figure 5a**. Following tumor inoculation on day 0, mice were administered intravenous injections of PBS, or MSN@Au on day 10. For the laser-treated groups, tumors were irradiated with an 808 nm laser (1 W/cm^2^, 5 min), which generated localized hyperthermia as monitored by thermal imaging (**Supplementary Figure 9**). Strikingly, the MSN@Au + laser cohort exhibited unparalleled tumor suppression, demonstrating ~90.0% tumor mass reduction compared to baseline. This efficacy significantly outperformed both the MSN@Au-only group (~20.8%) and Au NP + laser controls (~54.3%), as quantified by excised tumor weights and volumetric analysis (**Figures 5b–d**, **Supplementary Table S1**).

**Figure 6:**
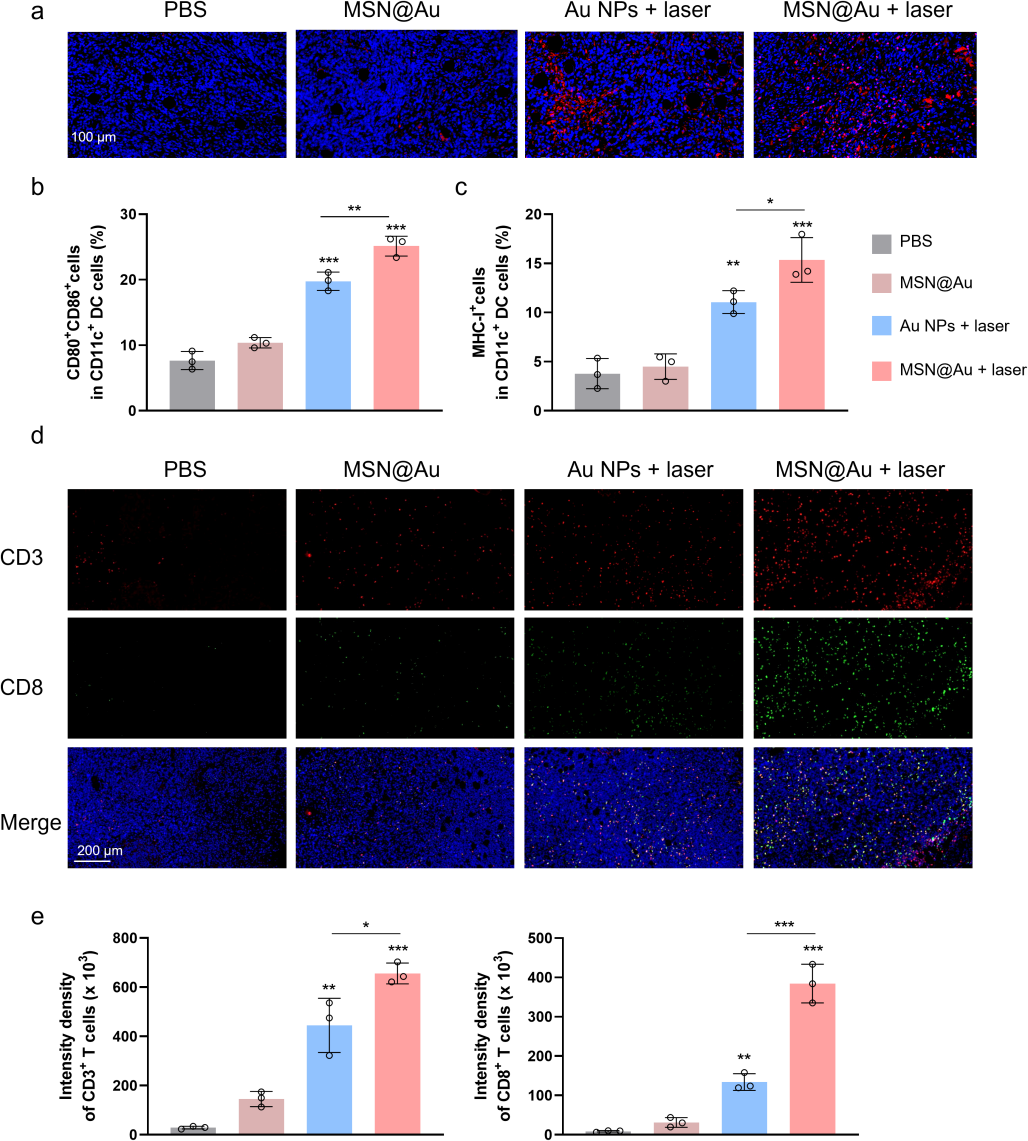
Immune regulation of MSN@Au-PTT combined therapy. **(a)** CRT expression levels across treatment groups. **(b)** Flow cytometry analysis of CD80^+^CD86^+^ DCs in tumor-draining lymph nodes. **(c)** Flow cytometry analysis of MHC I^+^ in DCs. **(d)** Immunofluorescence staining analysis of tumor-infiltrating lymphocytes: CD3^+^ T cell CD8^+^ T cell. **(e)** Quantification of CD3^+^ and CD8^+^ tumor-infiltrating T cells. ***P < 0.001, **P < 0.01, *P<0.05, calculated by one-way ANOVA.

To delineate the mechanistic of enhanced therapeutic efficacy, we performed multiparametric analysis of ICD biomarkers. High-resolution confocal imaging revealed laser-dependent membrane translocation of CRT, a cardinal “eat-me” signal for antigen-presenting cells, in MSN@Au + laser-treated tumors (**Figure 6a**). Quantitative analysis demonstrated 4.6-fold increased CRT translocation versus non-irradiated MSN@Au controls (**Supplementary Figure 3**), confirming that photothermally triggered cytosolic stress synergizes with nanoparticle-mediated drug delivery to amplify ICD initiation. The immunological cascade elicited by this strategy was further deconstructed through flow cytometry analysis. Remarkably, DC activation was substantially potentiated, as evidenced by an increase in CD80^+^CD86^+^ mature DC populations within tumor-draining lymph nodes (~25.1% in MSN@Au + laser, 19.7% in Au NPs + laser, 10.4% in MSN@Au alone, **Figure 6b**). This DC phenotypic maturation was accompanied by upregulated MHC class I expression (4.1-fold elevation versus PBS controls), suggesting enhanced antigen cross-presentation capacity critical for adaptive immune activation (**Figure 6c**). Spatiotemporal profiling of tumor-infiltrating lymphocytes (TILs) through immunofluorescence unveiled profound immune landscape remodeling. The combinatorial regimen induced massive lymphocyte recruitment, with CD3^+^ T cell percentages and CD8^+^ T densities lymphocytes (**Figures 6d, e**). The expression of IFN-γ further confirmed that the MSN@Au + laser promoted a markedly elevated level of CD8^+^CTL infiltration (**Supplementary Figure 11**). These quantitative histopathological metrics collectively validate successful conversion of immunologically inert (“cold”) neoplasms into lymphocyte-inflamed (“hot”) lesions through photothermal-chemically orchestrated ICD pathways.

**Figure 7:**
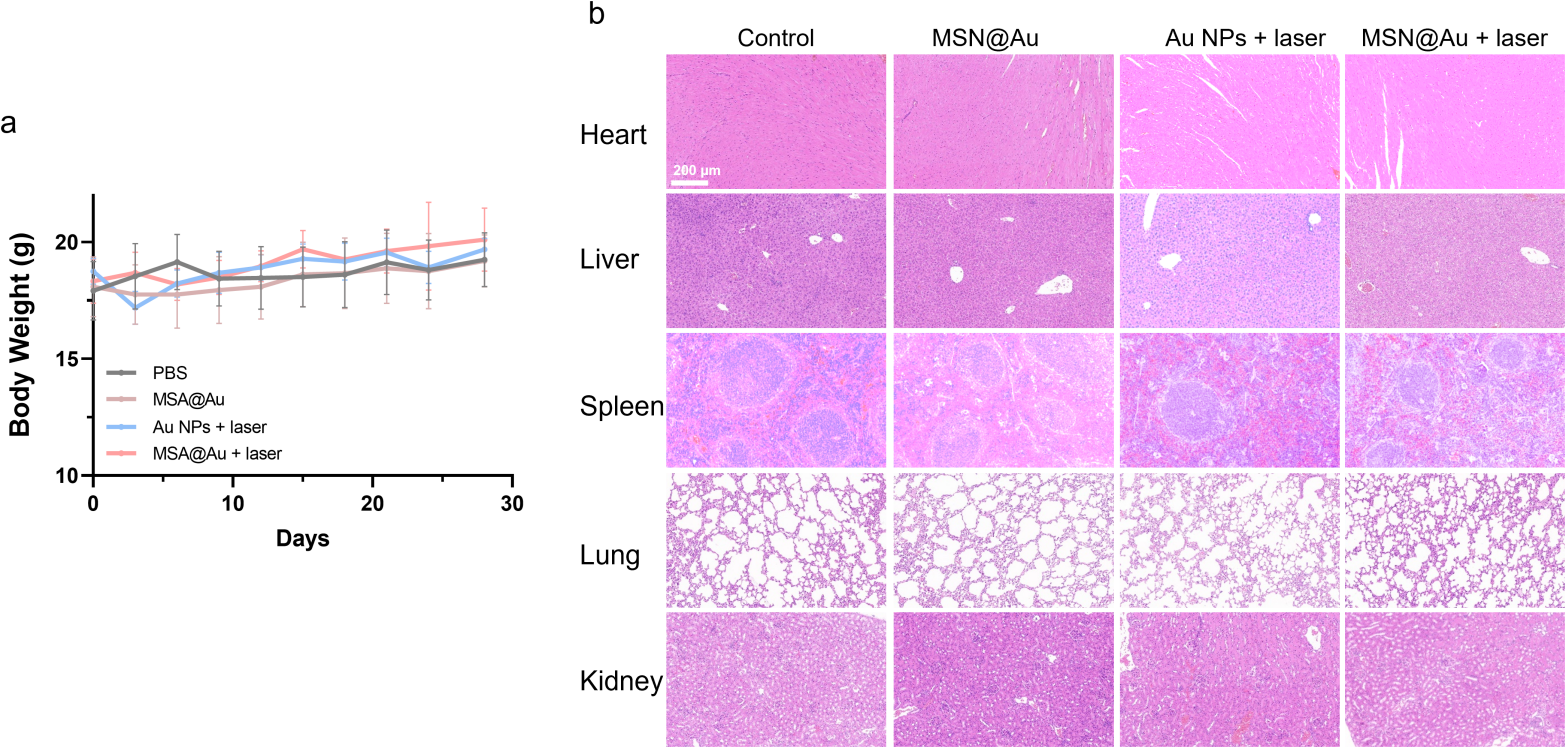
Biosafety of different treatments. **(a)** Body weight of mice in different groups during treatment period. **(b)** H&E staining of main organs (heart, spleen, liver, lung, and kidney).

The good biosafety of the composite nanosystem constitutes a fundamental prerequisite for its subsequent *in vivo* applications. Following intravenous injection, the weight of each group of mice was monitored every three days. Throughout the entire treatment phase, neither notable abnormal behaviors nor significant fluctuations in body weight were observed among the experimental groups (**Figure 7a**). To evaluate potential toxicity, major organs (including the heart, liver, spleen, lungs, and kidneys) were harvested for Hematoxylin and Eosin (H&E) staining. The histomorphological analysis of H&E-stained sections revealed no discernible pathology or abnormalities in either the control or experimental groups, underscoring that both Au NPs and MSN@Au exhibit negligible acute toxicity (**Figure 7b**). Furthermore, the results of blood biochemical analyses revealed no significant fluctuations in the parameters assessed across different treatment groups (**Supplementary Table S2**). Collectively, these comprehensive preliminary biosafety profiles, encompassing body weight monitoring, histopathological examination, and blood biochemical analysis, indicate that the Au-based composite nanosystem possesses exceptional biosafety. This finding establishes a solid foundation for its further application in both *in vivo* and *in vitro* studies.

## Conclusion

Our findings demonstrate that MSN@Au as a functionally integrated nanoplatform has several distinct advantages in cancer therapy. First, the nanocomposite exhibits excellent biocompatibility, with negligible cytotoxic effects observed in both MDA-MB-231 and 4T1 cells at concentrations up to 100 µg/mL under dark conditions. This favorable safety profile is critical for minimizing off-target effects in clinical applications. Second, MSN@Au’s significantly extended blood half-life (both α and β phases) compared to Au NPs positions it as a superior candidate for tumor-targeted drug delivery. Its design leverages prolonged circulation to maximize tumor accumulation while minimizing off-target effects, addressing critical limitations of conventional nanotherapies. Third, the synergistic integration of photothermal therapy enables precise tumor eradication, achieving a 50% reduction in cancer cell viability and a 90% tumor growth inhibition *in vivo* upon laser irradiation. The phototherapeutic effects are mechanistically supported by robust ROS generation and hyperthermia induction, which collectively disrupt cellular homeostasis. Finally, the platform’s ability to induce ICD represents a paradigm-shifting advantage. The upregulation of CRT exposure, coupled with DC maturation and T lymphocyte infiltration, confirms successful transformation of immunologically “cold” tumors into “hot” lesions. This immunomodulatory capacity positions MSN@Au as a promising candidate for tumor therapies.

However, several limitations warrant consideration. First, the underlying mechanisms driving the enhancement of photothermal conversion efficiency of MSN@Au, along with its long-term stability, warrant systematic evaluation. In addition, the absence of comparative data with existing nanoplatforms (e.g., gold nanorods or ICG-based systems) also hinders objective evaluation of therapeutic superiority. Then, while T cell infiltration metrics are promising, functional validation of antitumor immunity—such as tumor rechallenge experiments or cytokine profiling—would strengthen claims of durable immune memory. Optimization of laser delivery systems and comparative efficacy trials against benchmark nanomaterials will further refine the platform’s therapeutic potential.

## Data Availability

The raw data supporting the conclusions of this article will be made available by the authors, without undue reservation.
