# Supplementary material for: Mesoporous silica nanoparticles loaded Au nanodots: a self-amplifying immunotherapeutic depot for photothermal immunotherapy
Source: Front Immunol. 2025 Jun 18;16:1616539. doi: 10.3389/fimmu.2025.1616539 (PMC12213700; doi:10.3389/fimmu.2025.1616539)

**Supplementary Materials for**

**Mesoporous silica nanoparticles -loaded Au nanodots: A self- amplifying immunotherapeutic depot for photothermal immunotherapy**

**This PDF file includes:**

Figs. S1 to S11

Tables S1 to S2


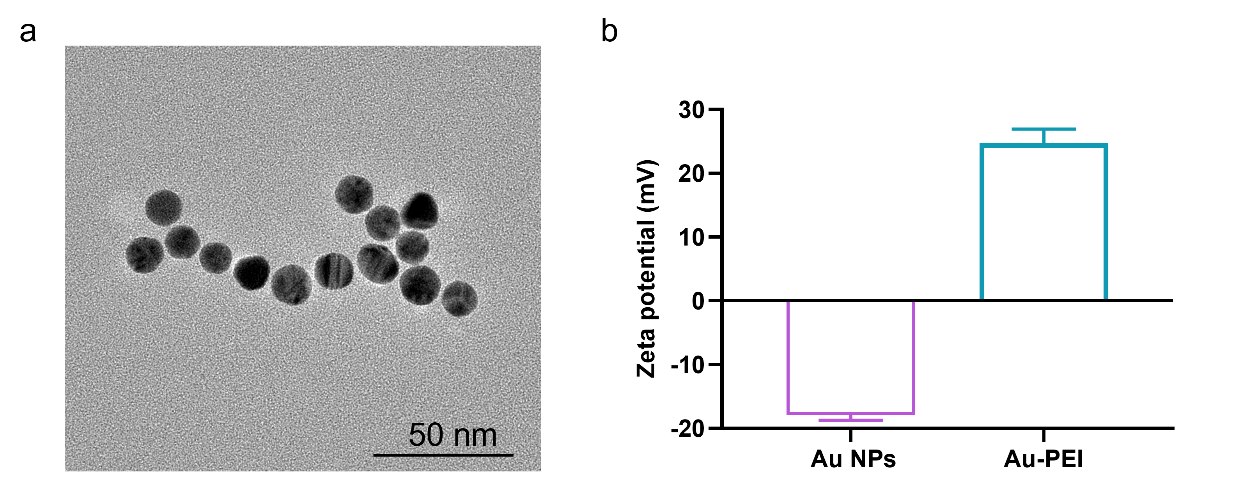


**Figure S1. a) TEM image of gold nanoparticles; b) Zeta potential plots of Au and Au-PEI.**


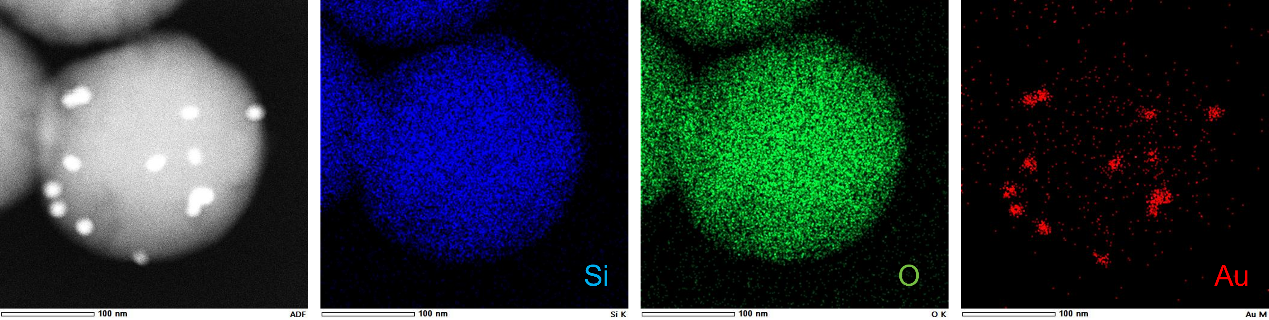


**Figure S2. Elemental mapping for Si, O, Au.**

**
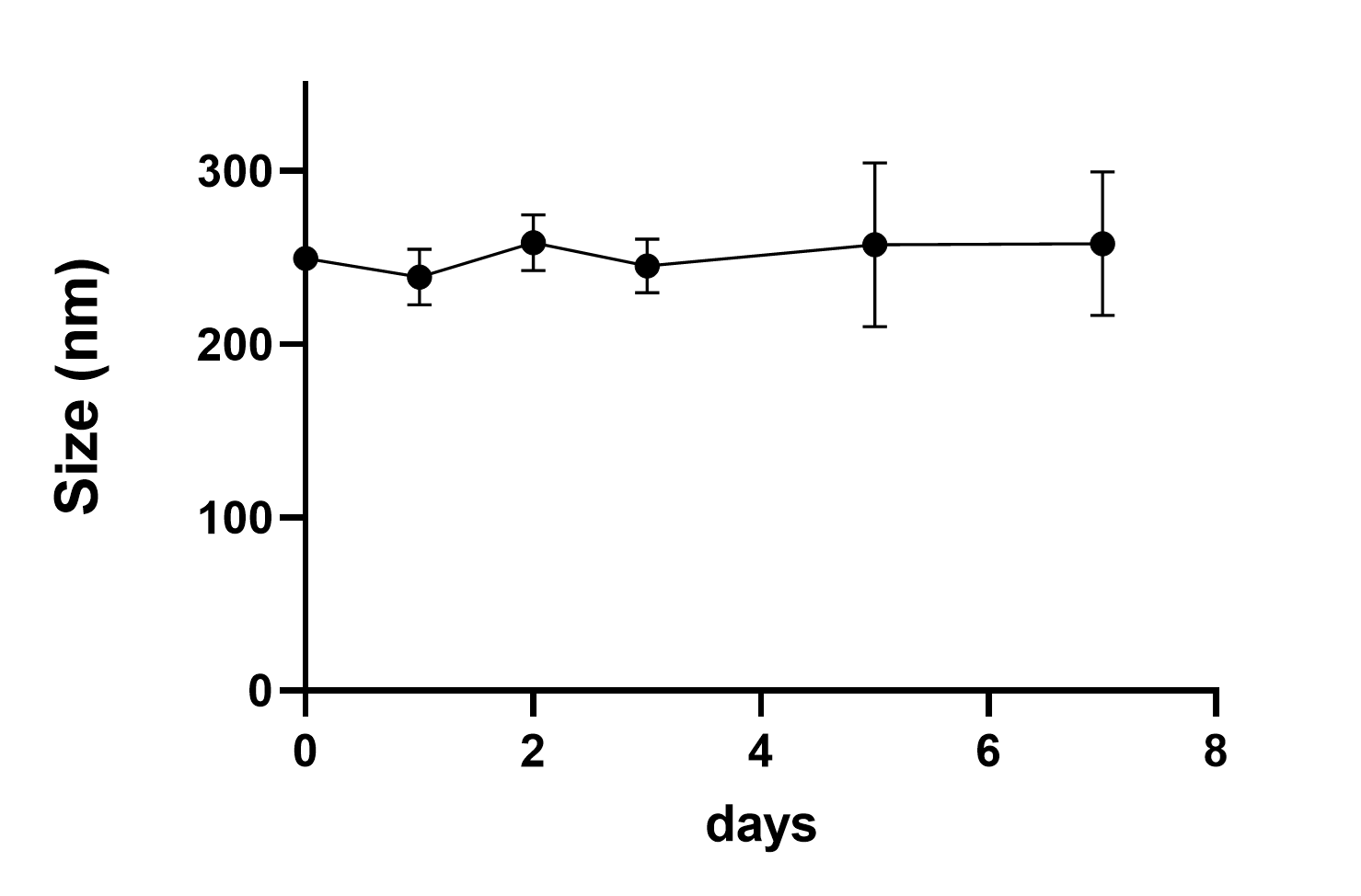
**

**Figure S3. Stability of MSN-Au in PBS during 7 days. The size of MSN-Au was measured by DLS.**

**
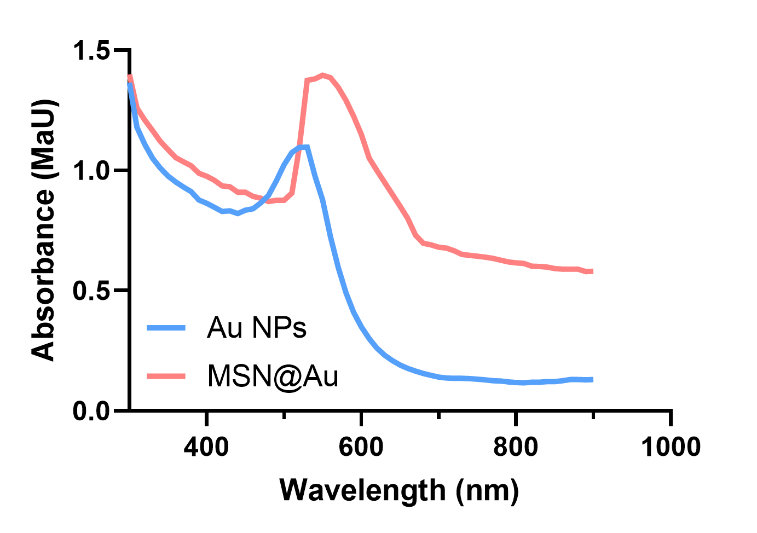
**

**Figure S4. Absorption analysis of Au NPs and MSN@Au at 100 μg/mL (Au content) by UV−vis spectroscopy.**


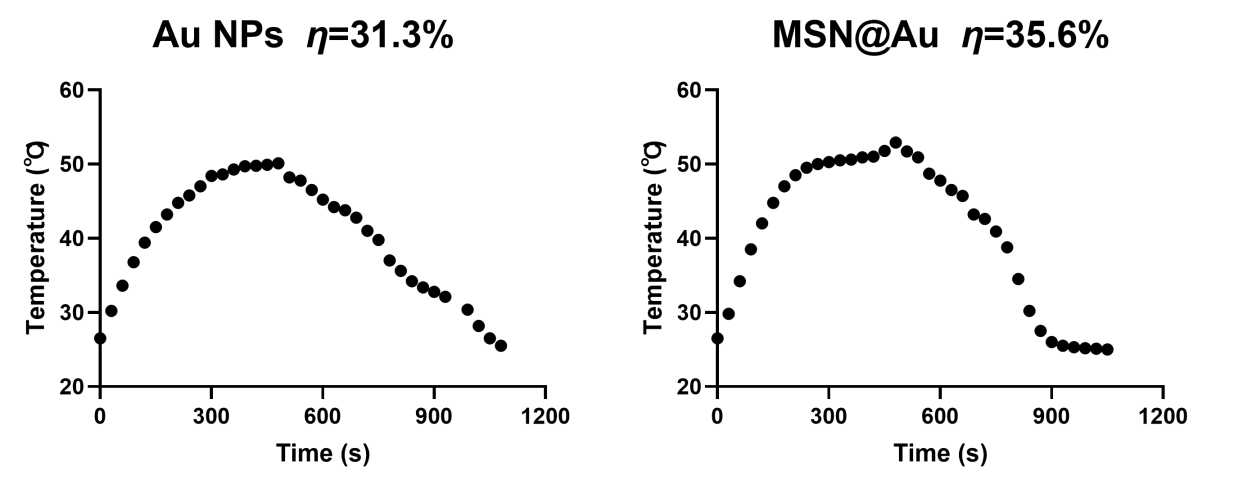


**Figure S5. Temperature change curves of Au NPs and MSN-Au (0.5 mg/mL of Au) under 808 nm laser (1.0 W/cm^2^) irradiation/cooling.**


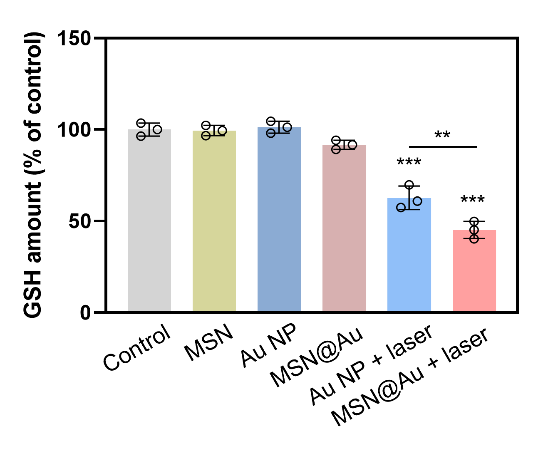


**Figure S6. GSH level of cells incubated with different treatments.**


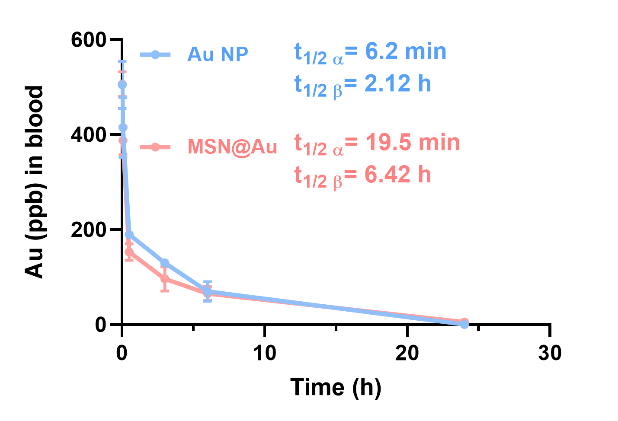


**Figure S7. The blood half-life of Au NPs and MSN-Au NPs.**


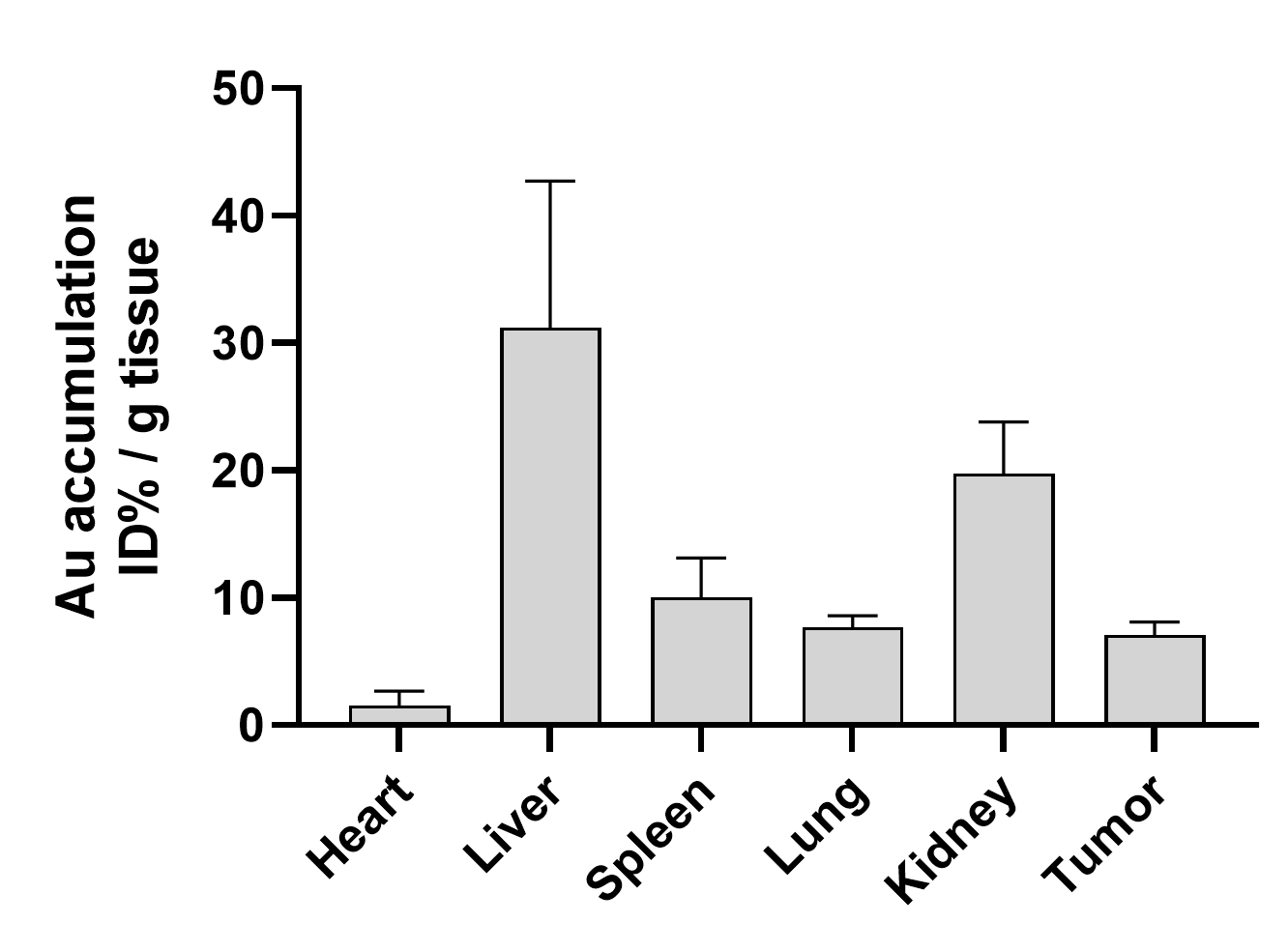


**Figure S8. Biodistribution of MSN@Au as determined by ICP-MS of the Au content at 24 h postinjection, expressed as % ID/g.**


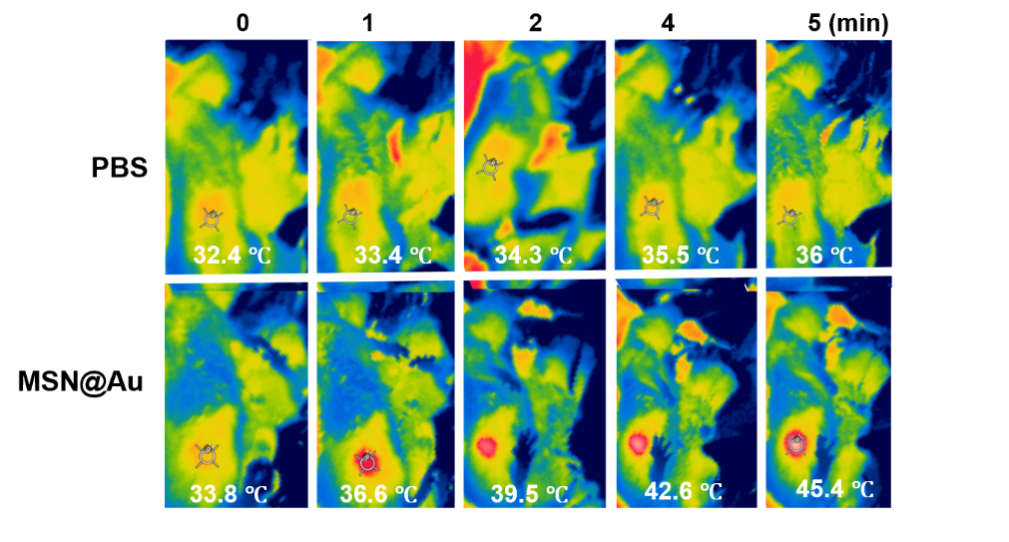


**Figure S9.** **Thermal imaging during the treatment process**


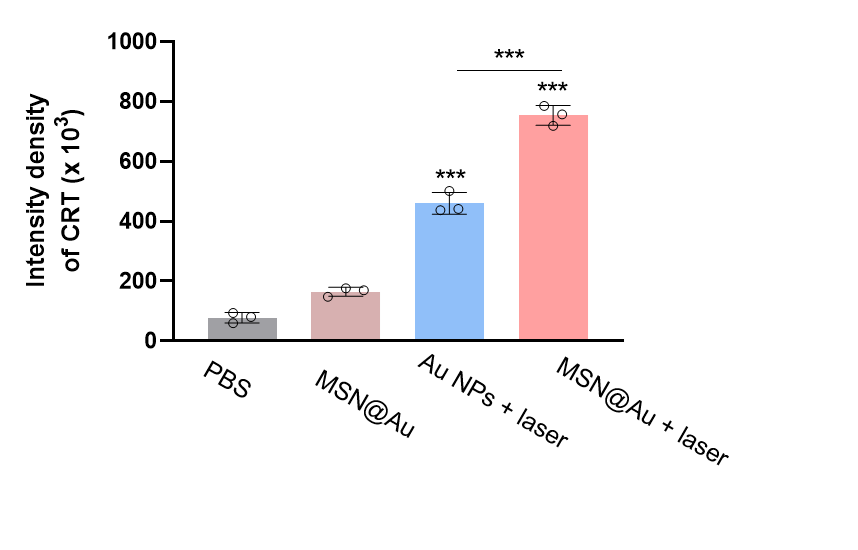


**Figure S10. Immunofluorescence staining analysis of CRT expression on tumor cells.**


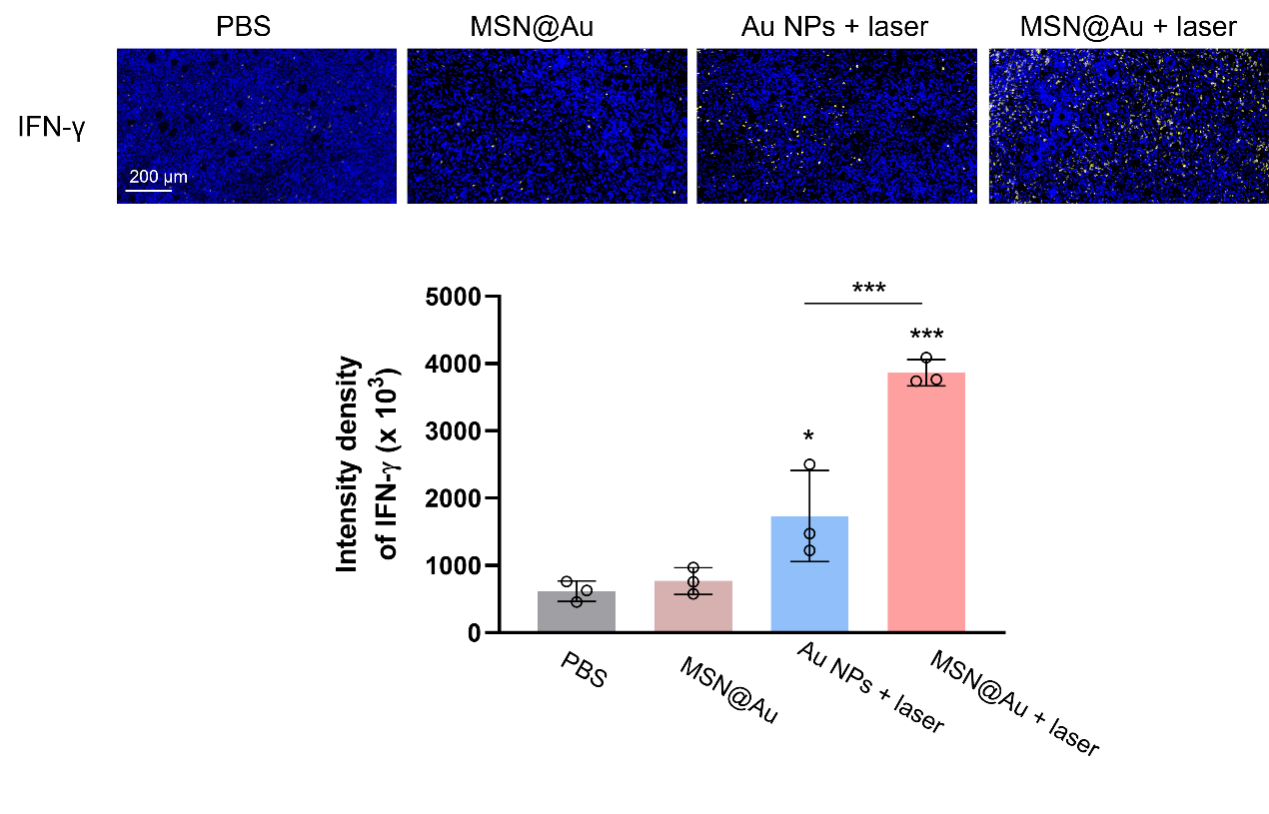


**Figure S11. Immunohistochemical staining of tumors for IFN-γ expression at day 28 after the indicated treatments.**

**Supplementary Table 1. Anti-tumor rate of different groups.**


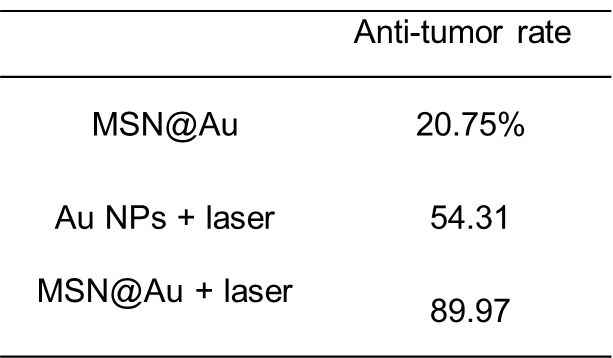


**Supplementary Table 2. Biochemical analyses of different groups.**


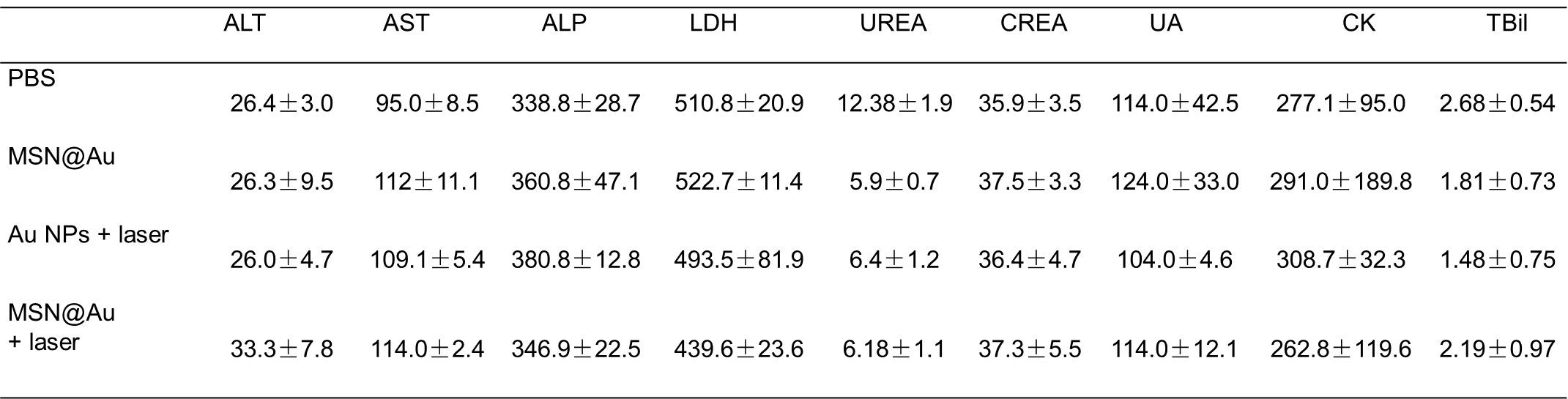

Supplement: Supplementary file 1 [file DataSheet1.docx]
